# Effects of pregnancy type, genotype, and parturition stage on ewe behavior

**DOI:** 10.5194/aab-69-289-2026

**Published:** 2026-05-12

**Authors:** Ömer Faruk Güngör, Necmettin Ünal, Yavuzkan Paksoy

**Affiliations:** 1 Department of Veterinary, Vocational School, Bolu Abant Izzet Baysal University, 14800 Mudurnu, Bolu, Türkiye; 2 Department of Animal Breeding and Husbandry, Ankara University Faculty of Veterinary Medicine, 06110 Ankara, Türkiye; 3 Department of Animal Breeding and Husbandry, Cukurova University Faculty of Ceyhan Veterinary Medicine, 01250 Adana, Türkiye

## Abstract

This study aimed to investigate the effects of pregnancy type, genotype, and parturition stage on sheep behavior. It also examined how pregnancy type and genotype influence labor characteristics. These evaluations were necessary because the length and difficulty of the early stages of parturition can impact the ewe's condition and parturition-related behavior, potentially disrupting the development of ewe–lamb bonding, which plays a critical role in neonatal lamb survival. Behavioral data were collected via video recordings from three sheep genotypes. The average lying and standing frequencies (hourly posture changes) during the first and second stages of parturition were 3.62 ± 0.33 and 8.53 ± 1.35, respectively ( P<0.001 ). In the first stage, these frequencies were 4.39 ± 0.51 for singleton pregnancies and 2.85 ± 0.38 for twin pregnancies ( P= 0.041). During the second stage, the frequencies were 12.64 ± 2.35 for singleton pregnancies and 4.42 ± 0.59 for twin pregnancies ( P= 0.013). The duration of the second stage of parturition was significantly affected by pregnancy type ( P= 0.009), averaging 48.18 ± 11.39 min for singleton pregnancies and 90.09 ± 10.09 min for twin pregnancies. Genotype had no significant effect on the duration of the second stage of parturition ( P= 0.782). Time of day had an impact ( P= 0.002), with 18:00–00:00 (Türkiye, UTC + 3) being the period with the highest proportion of births (41.56 %). In conclusion, parturition-related behaviors are generally influenced by pregnancy type and stage of labor, while genotype appears to have minimal effect. The lying and standing frequencies per hour may serve as a useful indicator for predicting pregnancy type and timing of delivery.

## 1 Introduction

Sheep breeding is a common branch of animal husbandry in semi-arid and arid regions of the world as sheep are able to graze and survive in these environments and produce yields on these lands where cattle often perform poorly. Therefore, sheep are considered to be an important livestock species in such regions (Akçapınar, 2000; Morris, 2009).

Behavioral traits in sheep can significantly influence production traits such as fertility, lamb mortality, growth, and meat quality (Ferguson and Warner, 2008; Pajor et al., 2008; Blache and Bickell, 2011; Gavojdian et al., 2015) Improving fertility and survival rates is essential for profitable livestock production (Buoio and Costa, 2020; Ungerfeld et al., 2021), and the survival rates of neonatal lambs are significantly affected by maternal behaviors (Nowak et al., 2000; Güngör et al., 2022). For instance, the majority of lamb mortality occurs within the first 3 d after birth (Nowak et al., 2000; Brien et al., 2010; Matheson et al., 2012), a period strongly influenced by the parturition process, particularly its duration and difficulty. The stages of parturition can affect the ewe's physical condition and parturition-related behavior, which are crucial for ewe–lamb bonding and, consequently, lamb survival (Flinn et al., 2020; Güngör and Ünal, 2020b; Güngör et al., 2022). In Australian flocks, approximately half of all lamb deaths are associated with prolonged parturition and related complications (Refshauge et al., 2016). Therefore, evaluating the first two stages of parturition is essential. Based on these findings, providing appropriate conditions tailored to pregnancy type and genotype is necessary to support both ewes and lambs during this critical period.

The parturition process in sheep consists of three stages. During the first stage (preparation for birth), ewes exhibit increased restlessness, and cervical dilation begins. This stage typically lasts between 3 and 6 h, although there is considerable variation. At this stage, the lamb tries to get into a suitable presentation for delivery, and ewes show slight or no visible signs. In the second stage (delivery of the fetus), ewes display clear abdominal contractions, and the cervix is fully dilated. This stage involves the expulsion of the lamb(s) and usually occurs within 30 min to 1 h. The third stage (expulsion of the placenta) occurs between the birth of the last lamb and the expulsion of the placenta, typically lasting 2 to 4 h (Jackson, 2004; NADIS, 2024). Throughout these stages, ewes exhibit various parturition-related behaviors, such as repeatedly lying down and standing up, scraping the foot on the ground, bleating, and licking areas where amniotic fluid has been spilled. The frequency of these behaviors increases as parturition progresses. Moreover, as pregnancy advances, feed intake tends to decrease due to increased uterine pressure (Ekesbo and Gunnarsson, 2018). Among these behaviors, the most observable and quantifiable traits are the lying and standing frequency, as well as the percentage of lying time. These behaviors were evaluated in this study because they can be easily monitored and recorded using accelerometer sensors.

Akkaraman, a fat-tailed native breed and the most common sheep breed in Türkiye, is known for its high survival rate, good growth performance, and strong adaptability to harsh conditions. Bafra, a thin-tailed synthetic breed (a stabilized composite breed developed by crossbreeding 75 % Chios and 25 % Karayaka), exhibits high reproductive performance, good adaptability, efficient milk production, and desirable meat quality (Akçapınar, 2000; Güngör et al., 2023). Crossbreeding between Bafra and Akkaraman has been carried out to combine the favorable traits of both breeds (Güngör and Ünal, 2020a; Güngör et al., 2022). These breed-specific traits may influence parturition-related behaviors and labor characteristics. Therefore, breed was included in the study to explore its impact on parturition-related behaviors and labor characteristics. Additionally, comparing genotypes enables the evaluation of how the genetic background affects these traits.

Though the lack of maternal instinct is known to result from factors such as dystocia, prolonged parturition length, and the negative effects of the first and second stages of parturition, the evaluation and comparison of parturition-related behaviors and labor traits in the first and second stage of parturition have not yet been studied together (Flinn et al., 2020; Güngör and Ünal, 2020b; Güngör et al., 2022; Redfearn et al., 2023;). This study hypothesized that pregnancy type (singleton vs. twin), genotype (Akkaraman, Bafra, BA F_1_), and parturition stage (first vs. second) significantly influence parturition-related behaviors in ewes. Specifically, it was predicted that twin pregnancies would be associated with longer labor durations and altered behavioral patterns, and it was specifically predicted that crossbred ewes (BA F_1_) would exhibit intermediate behavioral traits during parturition compared to their parent breeds, Akkaraman and Bafra. Accordingly, the aim of this study was to evaluate the effects of pregnancy type, genotype, and the first two stages of parturition on behavior and labor traits. The findings are expected to offer valuable insights for improving ewe husbandry practices by considering behavioral differences associated with pregnancy type and genotype.

## 2 Materials and methods

### 2.1 Ethical standards and study area

This study was approved by the Ankara University Animal Experiments Local Ethics Committee (reference no. 53184147-50.04.04/38558; Ankara, Türkiye) and was conducted at Gözlü State Farm (38°29^′^ N, 32°27^′^E; 1020 m altitude), which belongs to TİGEM, Ministry of Agriculture, in Konya Province, Central Anatolia region of Türkiye, where a steppe climate prevails.

### 2.2 Animals and housing conditions

A total of 52 multiparous ewes were used in the study. During the summer (July–September), the ewes grazed on wheat stubble. Flushing feeding was initiated 15–20 d before the mating period in September and continued during the first 30 d of mating. Mating was conducted by hand mating (controlled natural mating), artificial insemination, or pasture mating. Ewes were grazed on pasture until snowfall during early pregnancy but were not allowed to graze during late pregnancy and lambing. When grazing was not possible, roughage was provided during the first two-thirds of pregnancy. In the last one-third of pregnancy, each ewe received approximately 700 g d^−1^ of wheat in addition to roughage. Potable water was available ad libitum. At 3 d before expected lambing, ewes were moved to individual pens measuring 1.8 m^2^ ( 1.2×1.5 m, deep straw bedding), with feed and potable water within the comfort zone for sheep.

### 2.3 Experimental design

The lambing season occurred between February and March. The flock, consisting of approximately 1000 ewes, was monitored daily to identify ewes exhibiting signs of impending parturition. Additionally, some ewes had known lambing dates as they had been mated by hand mating (controlled natural mating) or artificially inseminated, and were moved to individual pens approximately 3 d before their expected lambing. The ear tag number and time of penning were recorded for each ewe before penning. During the penning period, all ewes were provided with feed and potable water. The data were collected during two consecutive lambing periods. In each period, a similar number of animals from each genotype was monitored, and no repeated measurements were taken from the same animals. All animals were managed under identical feeding and environmental conditions in accordance with the standard management practices of Gözlü State Farm. The climatic conditions in the barn (temperature and relative humidity) during the penning period were monitored. Humidity (50 %–80 %) and temperature (10–20 °C) were generally within the comfort zone for sheep breeding, which is defined to be 10–14 °C and 70 %–80 % relative humidity (Akçapınar, 2000).

The ewes in the study were fit, clinically healthy, and in good condition. They were not kept in the individual pen for more than 3 d. Some animals gave birth shortly after being placed in individual pens and some did not give birth within these 3 d; therefore, data from every ewe placed in the pens could not be included in the analysis. Behaviors during the first and second stages of parturition were compared. Therefore, it was necessary to have data from the same ewe in both stages to perform the comparison using a paired-sample t test. For this reason, data from 22 singleton-pregnant ewes (13 Akkaraman, 5 Bafra, 4 BA F_1_) and 22 twin-pregnant ewes (10 Akkaraman, 8 Bafra, 4 BA F_1_) were used for the behavioral comparison, although data from a total of 52 ewes (27 singletons and 25 twins; 28 Akkaraman, 14 Bafra, 10 BA F_1_) were used for the analysis of labor traits.

### 2.4 Behavioral observations and video analysis

Observations were carried out using a recording system consisting of a DVR and video cameras (Samsung SCO-2080RP). A total of six cameras were used, each positioned to monitor two adjacent lambing pens. As much as possible, all ewes were allowed to give birth without human intervention. However, lambing times were recorded as accurately as possible by the shepherds as this information was useful when reviewing the video footage, but efforts were made to minimize disturbance to the ewes during the observation process.

All video recordings were reviewed and coded by the first author, who systematically analyzed the footage to identify and quantify parturition-related behaviors and labor traits based on the predefined ethogram. Data on the behavior of the ewes were collected during the two observation stages. The beginning of the second stage of parturition was considered to be the first obvious abdominal contractions (labor pains, restlessness) preceding delivery. The first stage was defined as the 3 h period prior to the second stage due to the subtle or absent signs during this phase, making the onset of this stage impossible to determine by direct observation.

### 2.5 Ethogram and definitions

The ethogram of all behaviors and labor traits observed in this study is provided in Table 1. The assisted lambing in this study was conducted by the shepherd, who monitored the ewes during parturition without disturbing them and did not intervene unless necessary. The lambing process was allowed to proceed naturally as much as possible, but when assistance was required, the shepherd provided only the necessary help. These interventions were generally limited to cases where labor exceeded 1 h after its onset and were deemed to be necessary. No births in the study required intervention by a veterinarian. Thus, the births occurred either with assistance or without assistance. The birth times were divided into four equal 6 h intervals within a 24 h period, which were 06:00–12:00 (all times are local time, Türkiye, UTC +3 ), 12:00–18:00, 18:00–00:00, and 00:00–06:00.

**Table 1:** Ethogram of all behaviors and labor traits used in the study.

Number of stand-ups: count of times the animal stood up and stayed up for ≥ 5 s.
Number of lie-downs: count of times the animal lay down and stayed down for ≥ 5 s.
Lying/standing frequency (h^−1^): hourly count of posture changes (based on the first two metrics).
Lying time (min): duration spent lying down.
Lying time (%): proportion of time spent lying down (based on lying time).
Length of second stage of parturition (min): start of the second stage and expelling the last lamb.
Time until the first birth: for twin births, time between start of second stage and first lamb expulsion.
Time between the first and second lamb births: the time interval between first and second lamb expulsion.
Delivery position: the position of the ewe (standing or lying) during the lamb expulsion.
Lambing assistance: assisted (applied by shepherd) or unassisted.
Birth time: exact time of lamb expulsion and a day categorized into four intervals.

### 2.6 Data analysis

The data were analyzed using SPSS software (SPSS, 2013). Differences between stages in the lying and standing frequency per hour (hourly posture changes) and in the percentage of lying time were analyzed using the paired-sample t test to compare the two stages of parturition. The effects of pregnancy type and genotype on the lying and standing frequency per hour and on the percentage of lying time were analyzed using the general linear model (GLM) procedure with Tukey's honestly significant difference (HSD) test. The effects of pregnancy type and genotype on the duration of the second stage of parturition were also analyzed using the GLM procedure in SPSS. The effects of genotype on the time to the first lamb and the duration between the first and second births were analyzed using one-way ANOVA with Tukey's HSD test using data from twin-pregnant ewes only as these temporal traits are defined exclusively in twin births. The statistical significance of differences in categorical labor traits (presented as percentages) was assessed using the $\chi^{2}$ test. The alpha level was set at 0.05.

## 3 Results

### 3.1 Effects of pregnancy type, genotype, and parturition stage on ewe behaviors

#### 3.1.1 Effects of the factors on lying and standing frequency

**Figure 1:**
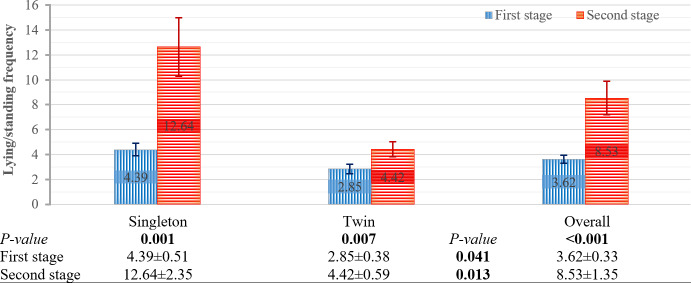
Effect of pregnancy type and parturition stage on lying and standing frequency (h^−1^). Means ± SE. Bars show means with SE bars. SE: standard error.

**Figure 2:**
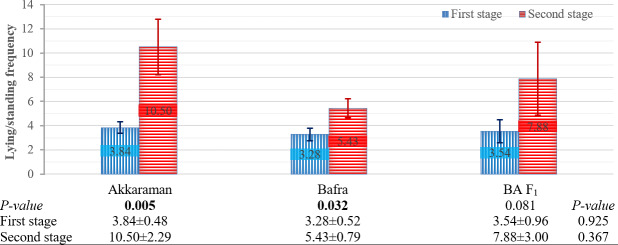
Effect of genotype and parturition stage on lying and standing frequency (h^−1^). Means ± SE. Bars show means with SE bars. SE: standard error. BA F_1_: Bafra × Akkaraman F_1_.

The effects of pregnancy type, genotype, and stage on lying and standing frequency per hour are presented in Figs. 1 and 2. Pregnancy type had a significant effect on this frequency at both stages of parturition (Fig. 1) (first and second stages: P= 0.041 and P= 0.013, respectively), whereas genotypes had no significant effect at either stage (Fig. 2). Significant differences in these frequencies between parturition stages were observed for singleton pregnancies, twin pregnancies, and the overall score (Fig. 1) ( P= 0.001, P= 0.007, and P<0.001 , respectively). Additionally, these differences were significant across genotypes (Fig. 2) ( P= 0.005 for Akkaraman and P= 0.032 for Bafra), except for the BA F_1_ genotype, which approached significance ( P= 0.081).

#### 3.1.2 Effects of the factors on the percentage of lying time

**Figure 3:**
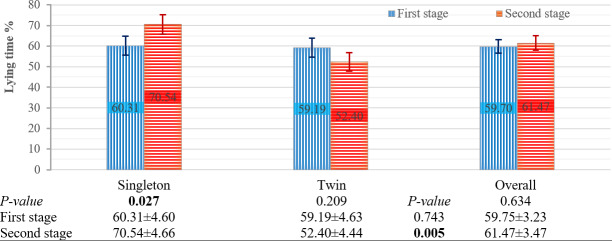
Effect of pregnancy type and parturition stage on percentage of lying time. Means ± SE. Bars show means with SE bars. SE: standard error.

**Figure 4:**
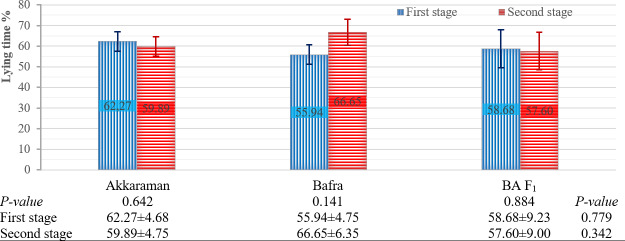
Effect of genotype and parturition stage on percentage of lying time. Means ± SE. Bars show means with SE bars. SE: standard error. BA F_1_: Bafra × Akkaraman F_1_.

The effects of pregnancy type, genotype, and stage on the percentage of lying time are presented in Figs. 3 and 4. Pregnancy type had a significant effect on this percentage during the second stage ( P= 0.005), while the stage of parturition affected this trait in singleton ewes ( P= 0.027) (Fig. 3). No other factors had a significant effect on the percentage of lying time.

The interaction effects of pregnancy type and genotype on the lying and standing frequency per hour were not significant in either stage ( P= 0.984 and P= 0.409 for the first and second stages, respectively). Similarly, their interaction had no significant effect on percentage of lying time ( P= 0.374 and P= 0.788 for the first and second stages, respectively).

### 3.2 Labor traits

The time durations (min) of the second stage of parturition according to pregnancy type and genotype are presented in Table 2. Twin-pregnant ewes exhibited significantly longer durations ( P= 0.009) in the second stage of parturition compared to single-pregnant ewes. Although Akkaraman ewes had longer durations in this stage, the differences among genotypes were not statistically significant.

**Table 2:** Length of the second stage of the parturition (min).

Items	Mean ± SE
Pregnancy type	
Singleton	48.18 ± 11.39
Twin	90.09 ± 10.09
Genotype	
Akkaraman	75.69 ± 9.35
Bafra	66.69 ± 13.31
BA F_1_	65.04 ± 16.01
Overall	68.05 ± 7.81
P value	
Pregnancy type	**0.009**
Genotype	0.782
Pregnancy type ^*^ genotype	0.079

SE: standard error; BA F_1_: Bafra × Akkaraman F_1_. Bold values indicate statistically significant differences ( P<0.05 ). ^*^: indicates an interaction between factors.

The time to the first lamb (min) and the interval between the first and second lamb in twin-pregnant ewes, categorized by pregnancy type and genotype, are presented in Table 3. Akkaraman ewes exhibited longer durations for both the time to the first lamb and the interval between lambs; however, these differences were not statistically significant compared to other genotypes.

**Table 3:** Time until first birth and interval between births in twin pregnancies (min).

Items	Mean ± SE
The time until the first birth	
Akkaraman	50.49 ± 12.44
Bafra	33.67 ± 7.03
BA F_1_	26.03 ± 7.05
P value	0.293
Overall	39.54 ± 6.34
The duration between first and second births	
Akkaraman	67.59 ± 14.23
Bafra	43.44 ± 10.93
BA F_1_	49.06 ± 3.33
P value	0.350
Overall	55.19 ± 7.55

SE: standard error; BA F_1_: Bafra × Akkaraman F_1_.

**Figure 5:**
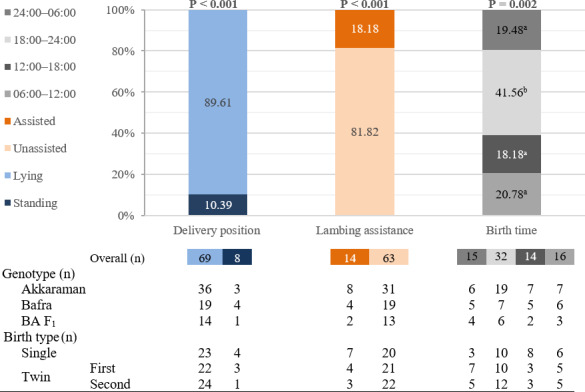
Overall results of the lambing traits. BA F_1_: Bafra × Akkaraman F_1_.

The position of the ewe during lamb expulsion, assistance requirements, and birth time results for different lamb genotypes are presented in Table 4 and Fig. 5. Akkaraman ewes showed a significantly higher proportion of nighttime lambing (18:00–06:00; P= 0.042). Additionally, twin-pregnant ewes were more likely to give birth at night ( P= 0.011). Other differences presented in Table 4 were not statistically significant.

**Table 4:** Effects of genotype and birth type on lambing traits (%).

Items		Born while the	Born	Birth time			
		ewe is lying	unassisted	06:00–18:00	18:00–06:00	P value	
Genotype							
Akkaraman		92.31	79.49	35.90	64.10	**0.042**	
Bafra		82.61	82.61	47.83	52.17	0.768	
BA F_1_		93.33	86.67	33.33	66.67	0.068	
Birth type							
Single		85.19	74.07	51.85	48.15	0.785	
Twin	First	88.00	84.00	32.00	68.00	**0.011**	
	Second	96.00	88.00	32.00	68.00	**0.011**	
P -value							
Genotype		0.419	0.823	0.573			
Birth type		0.420	0.404	0.234			

Birth-time was grouped into two complementary periods; changes in one category necessarily imply changes in the other. Therefore, the chi-square test provides a single p-value for the day–night comparison. Bold values indicate statistically significant differences ( P<0.05 ). BA F1: Bafra × Akkaraman F1.

Although genotype subgroup sizes were relatively small, genotype-specific tendencies were presented in Table 4 using two time periods, whereas the overall distribution was provided in Fig. 5 using four time periods. Additionally, birth time analysis was simplified into day–night periods, as commonly applied in the literature to enhance interpretability.

Birth time was grouped into two complementary periods, with changes in one category necessarily implying changes in the other. Therefore, the $\chi^{2}$ test provides a single p value for the day–night comparison. BA F_1_ denotes Bafra × Akkaraman F_1_

In the overall results (Fig. 5), the percentage of lambs born while the ewe was lying down (89.61 %) was significantly higher ( P<0.001 ) than the percentage born while the ewe was standing (10.39 %). Similarly, the percentage of lambs born unassisted (81.82 %) was significantly higher ( P<0.001 ) than that of those requiring assistance (18.18 %). Additionally, the differences in the distribution of lambing times were statistically significant ( P= 0.002), and the proportion of births was higher during 18:00–00:00 than in each of the other three periods (06:00–12:00, 12:00–18:00, 00:00–06:00), while these three periods did not differ from one another.

## 4 Discussion

### 4.1 Effects of pregnancy type, genotype, and parturition stage on ewe behaviors

Although some parturition behaviors have been investigated using accelerometer sensors (Fogarty et al., 2020; Smith et al., 2020; Gurule et al., 2021; Sohi et al., 2022), these sensor-based studies have described behavioral changes around lambing, but they have not parsed these patterns by pregnancy type or genotype, nor have they contrasted the first and second stages of parturition. Therefore, these findings could not be efficiently compared in this study. For example, Fogarty et al. (2020) showed pronounced increases in walking and posture change frequency at lambing using ear tag accelerometers, indicating greater restlessness on the day of parturition. Similarly, Sohi et al. (2022) classified grazing, rumination, licking, walking, and idling with high concordance to visual observation and predicted lambing time up to ∼10 d in advance via machine learning models. Complementing these, Smith et al. (2020) used tri-axial accelerometers to detect activity distribution shifts associated with lambing. Additionally, Gurule et al. (2021) showed that direct sensor metrics increased 1–2 h pre-lambing. Taken together, these studies establish robust sensor-based changes around lambing while underscoring the gap the present study addresses by comparing behaviors across pregnancy types and genotypes and explicitly contrasting the first versus second stages of parturition.

#### 4.1.1 Effects of pregnancy type

Pregnancy type had a significant effect on the lying- and standing-frequency behavior during both the first and second stages of parturition, with singleton ewes showing higher frequencies than twin ewes. These observed differences in this behavior may be considered when evaluating pregnancy type.

In the first stage of parturition, pregnancy type had no significant effect on the percentage of lying time, although pregnancy type significantly affected the lying- and standing-frequency behaviors. This is because the percentage of lying time was similar between singleton and twin ewes in the first stage; however, singleton ewes showed significantly higher lying and standing frequency compared to twin ewes in this stage. This difference, which was absent in terms of the percentage of lying time, may be attributed to the greater amount of amniotic fluid, placentas, and lambs in twin-pregnant ewes, which makes the lying- and standing-frequency behavior more difficult. This increased load raises abdominal pressure and physical discomfort, thereby making frequent posture changes more difficult for twin-pregnant ewes.

In the second stage of parturition, pregnancy type had a significant effect on both the percentage of lying time and the lying- and standing-frequency behaviors. In twin-pregnant ewes, both the percentage of lying time and the lying- and standing-frequency behaviors were lower than those of singleton ewes. Although the ewe's position during lamb expulsion was predominantly lying and although it would be expected that twin-pregnant ewes would spend more time lying down during the second stage than singleton ewes, the findings of this study did not support this expectation. This discrepancy may be explained by more pronounced uterine contractions in twin pregnancies, which are associated with greater pain and discomfort. These factors could limit the percentage of lying time and reduce the percentage of lying time behaviors compared to singletons in the second stage. These results indicate a significant behavioral difference between singleton- and twin-pregnant ewes during the second stage of parturition, indicating that pregnancy type influences both the percentage of lying time and the lying- and standing-frequency behaviors at this stage.

#### 4.1.2 Effects of genotype

Although genotype had no significant effect on the lying- and standing-frequency behaviors during either stage, Akkaraman ewes showed numerically higher lying and standing frequency than other genotypes, particularly during the second stage of parturition. This numerically higher frequency appears to suggest that Akkaraman ewes were more affected by the second stage of parturition. As expected, the lying- and standing-frequency behaviors in the crossbred BA F_1_ ewes (50 % Akkaraman × 50 % Bafra) were intermediate between the Akkaraman and Bafra genotypes.

In both stages of parturition, the genotype had no significant effect on the percentage of lying time, and these percentages for each genotype during the first and second stages of parturition were relatively similar. Overall, the total time spent lying and standing during these stages was approximately 60 % and 40 %, respectively. While the difference was not significant, it is noteworthy that the crossbred BA F_1_ genotype (50 % Akkaraman × 50 % Bafra) showed an intermediate percentage of lying time between Akkaraman and Bafra in the first and second stages, as expected.

#### 4.1.3 Effects of parturition stage

The stages of parturition significantly affected the lying- and standing-frequency behaviors. This significant difference was observed across singleton-pregnant, twin-pregnant, Akkaraman, and Bafra ewes. In contrast, the difference between parturition stages was not statistically significant for BA F_1_ ewes, although the P value was close to the threshold for significance. Overall, the lying- and standing-frequency behaviors approximately doubled during the second stage of parturition compared to the first stage. The increase in the lying- and standing-frequency behaviors during the second stage compared to during the first stage was expected as the approaching delivery causes increased restlessness. These changes in this behavior could be taken into account as a possible factor when assessing the approach of birth. Twin-pregnant ewes exhibited approximately twice the lying- and standing-frequency behavior during the second stage compared to during the first stage. Similarly, singleton-pregnant ewes showed a 3-fold increase in this behavior during the second stage. These observed differences in behavioral patterns may be considered when evaluating pregnancy type. When the second stage of parturition was evaluated separately, singleton pregnant ewes exhibited 3 times more lying- and standing-frequency behavior than twin-pregnant ewes. These findings were consistent with those of Özdemir and Altin (2007), who studied Karya sheep and reported that singleton-pregnant ewes displayed four lying and standing behaviors in 15 min, while twin-pregnant ewes exhibited six lying and standing behaviors in 90 min. The stage of parturition significantly affected the percentage of lying time in singleton-pregnant ewes, with the percentage during the first stage being significantly lower than that during the second stage. In contrast, twin-pregnant ewes showed a numerically higher percentage of lying time during the first stage compared to the second stage. These results indicate that the stage of parturition significantly influences lying behavior in singleton-pregnant ewes, whereas twin-pregnant ewes show relatively stable lying patterns across stages. This appears to suggest that behavioral responses to the progression of parturition differ between pregnancy types. In addition, the stage of parturition had no effect on the percentage of lying time in the genotypes. Notably, the percentage of lying time numerically increased during the second stage for Bafra ewes, whereas it remained relatively unchanged for Akkaraman and BA F_1_ ewes. According to these results (Fig. 4), it can be inferred that Akkaraman and BA F_1_ ewes were more affected by the second stage of parturition compared to Bafra ewes.

### 4.2 Labor traits

The duration of the second stage of parturition was not affected by genotype but was influenced by pregnancy type. The duration observed in twin-bearing ewes was approximately twice that of singleton-bearing ewes, as expected due to the nature of multiple births. Similarly, Cloete (1992) and Özdemir and Altin (2007) reported a significant effect of pregnancy type on the duration of the second stage of parturition. Although Cloete (1992) and Dwyer and Lawrence (1998) found a significant genotype effect on this stage, such an effect was not observed in the present study. In twin-bearing ewes in this study, the time until the birth of the first lamb was approximately 40 min, and the interval between the births of the first and second lambs was about 55 min. These durations were not affected by genotype, but there was considerable variation between them. This finding was consistent with the results of Dwyer and Lawrence (1998).

The duration of the second stage of parturition for single-bearing Corriedale and twin-bearing Merino ewes was reported to be 37 and 92 min, respectively (Freitas-De-Melo et al., 2017; Flinn et al., 2020). In the study by Freitas-De-Melo et al. (2017), this duration was defined as the interval from the appearance of the fetal forelimbs or hindlimbs (extremities) to the expulsion of the second lamb. In contrast, Flinn et al. (2020) defined it as the interval from the appearance of fluids or the sac at the vulva to the expulsion of the second lamb. A comparison of these two studies with the present findings revealed that the duration of the second stage of parturition for single-bearing ewes was approximately 10 min longer than that reported by Freitas-De-Melo et al. (2017), whereas the result for twin-bearing ewes closely aligned with the findings of Flinn et al. (2020). The discrepancy between the present study and that of Freitas-De-Melo et al. (2017) for single-bearing ewes can be attributed to differences in the criteria used to define the onset of the second stage. In the present study, the first visible abdominal contractions prior to delivery were considered to be the beginning of the second stage, which is partially consistent with the definition used by Flinn et al. (2020), and this may explain the similarity in the results. In this study, Akkaraman ewes are fat-tailed sheep; Bafra ewes are a long- and thin-tailed breed; BA F_1_ ewes are long- and thin-tailed, but they have fat accumulation at the tail root. Therefore, the observation of fluids or sacs at the vulva with the camera is difficult at the beginning of the second stage of parturition in these ewes. Hence, the first obvious abdominal contractions before delivery were evaluated as signs of the beginning of the second stage of parturition in this study. Additionally, the duration of the second stage of parturition was reported to be highly variable among breeds such as Blackface ewes (62 or 41 min), Suffolk ewes (84 or 41 min), twin-bearing Karya ewes (90 minutes), single-bearing Karya ewes (15 min), twin-bearing Çine Çaparıewes (43 min), single-bearing Çine Çaparıewes (29 minutes), Dormer ewes (67 min), and Mutton Merino ewes (92 min). (Cloete, 1992; Dwyer and Lawrence, 1998, 2000; Özdemir and Altin, 2007). The beginning of the second stage of parturition was evaluated differently in these articles, for instance, in the studies of Dwyer and Lawrence (the appearance of fluid or a sac at the vulva), Altın et al. (the first discomfort behaviors or abdominal contractions), and Cloete (first definite sign). Accordingly, variation in results among these studies can be attributed to differences in the breeding methods, the condition of the animals, and the different assessments of the onset of this stage.

When the time of day was evaluated (06:00–18:00 as daytime and 18:00–06:00 as nighttime), Akkaraman, BA F_1_, and twin-bearing ewes showed a preference for lambing during the night, whereas Bafra and single-bearing ewes were not affected by the time of day. The preference of twin-bearing ewes for nighttime lambing may be attributed to the longer and more uncomfortable nature of their deliveries. In contrast, the lack of time-of-day effect in Bafra ewes may be explained by their genetic background because the Bafra genotype has 75 % Chios genotype, which is a garden sheep rearing closer to humans (Akçapınar, 2000).

The number of ewes that gave birth between 18:00 and 00:00 was significantly higher (41.56 %) than during the other three periods (00:00–06:00, 06:00–12:00, and 12:00–18:00) (Fig. 5). Overall, 80 % of births occurred between 06:00 and 00:00. Dwyer and Lawrence (1998) reported that lambs showed a slight tendency to be born during the day (06:00–18:00) rather than overnight. For Blackface ewes, significantly more lambs were born between 12:00 and 18:00 (36.9 %), and significantly fewer were born between 00:00 and 06:00 (15.4 %). For Suffolk ewes, the pattern was similar but not statistically significant (31.9 % vs. 19.4 %). The findings of this study are similar to those reported by Dwyer and Lawrence (1998) in terms of fewer births occurring during the overnight period. In this study, lambing assistance was required in 18.18 % of births overall, with rates of 21 % for Akkaraman, 17 % for Bafra, and 13 % for BA F1 ewes. These differences among genotypes were not statistically significant. These rates were generally within the range reported for Suffolk (25 %) and Blackface (11 %) (Dwyer and Lawrence, 1998) but were higher than the 7 % reported for Merino ewes (Flinn et al., 2020). Overall, when the labor traits are evaluated as a whole, most births occurred between 18:00 and 00:00, and twin pregnancies were consistently associated with longer labor durations, thereby highlighting the need for closer monitoring in such situations.

## 5 Conclusions

Pregnancy type and parturition stage significantly affected the lying and standing frequency per hour in ewes. Singleton pregnancies were associated with higher lying and standing frequencies per hour compared to twin pregnancies in both stages, and these frequencies increased markedly during the second stage. Additionally, singleton ewes exhibited significantly greater percentage of lying time in the second stage than twin ewes. Genotypes had no significant effect on these behaviors. These findings highlight that behavioral patterns during parturition are primarily influenced by pregnancy type and parturition stage rather than genotype. Most lambing occurred while the ewe was lying, without assistance, and predominantly between 18:00 and 00:00. In conclusion, pregnancy type was associated with differences in lying and standing frequency in both stages and the proportion of time spent lying in the second stage.

## Data Availability

The datasets presented in this study are available from the corresponding author upon request.
